# Clinicopathological and molecular differences between stage IV screen-detected and interval colorectal cancers in the Flemish screening program

**DOI:** 10.3389/fonc.2024.1409196

**Published:** 2024-09-02

**Authors:** Isabelle Neefs, Thuy Ngan Tran, Allegra Ferrari, Sharon Janssens, Koen Van Herck, Ken Op de Beeck, Guy Van Camp, Marc Peeters, Erik Fransen, Sarah Hoeck, Guido Van Hal

**Affiliations:** ^1^ Center of Medical Genetics, University of Antwerp and Antwerp University Hospital (UZA), Edegem, Belgium; ^2^ Center for Oncological Research (CORE), University of Antwerp and Antwerp University Hospital (UZA), Antwerp, Belgium; ^3^ Research group on Social Epidemiology and Health Policy, Department of Family Medicine and Population Health (FAMPOP), University of Antwerp, Antwerp, Belgium; ^4^ Centre for Cancer Detection, Bruges, Belgium; ^5^ Department of Health Sciences (DISSAL), University of Genoa, Genoa, Italy; ^6^ Belgian Cancer Registry, Brussels, Belgium

**Keywords:** colorectal cancer, clinicopathological differences, interval cancer, molecular alterations, screening

## Abstract

**Introduction:**

Interval cancer (IC) is an important quality indicator in colorectal cancer (CRC) screening. Previously, we found that fecal immunochemical test (FIT) ICs are more common in women, older age, right-sided tumors, and advanced stage. Here, we extended our existing stage IV patient cohort with clinicopathological and molecular characteristics, to identify factors associated with FIT-IC.

**Methods:**

Logistic regression models were fit to identify variables associated with the odds of having a stage IV FIT-IC. Multivariate models were corrected for gender, age, and location.

**Results:**

A total of 292 screen-detected (SD) CRCs and 215 FIT-IC CRCs were included. FIT-IC CRC had 5 fold higher odds to be a neuroendocrine (NET) tumor and 2.5 fold higher odds to have lymphovascular invasion. Interestingly, some variables lost significance upon accounting for location. Thus, tumor location is a critical covariate that should always be included when evaluating factors related to FIT-IC.

**Conclusions:**

We identified NETs and lymphovascular invasion as factors associated with increased odds of having a stage IV FIT-IC. Moreover, we highlight the importance of tumor location as a covariate in evaluating FIT-IC related factors. More research across all stages is needed to clarify how these insights might help to optimize the Flemish CRC screening program.

## 1 Introduction

Colorectal cancer (CRC) is one of the leading causes of cancer-related deaths worldwide. In Belgium, CRC is the third most common cancer in men and second most common in women (1). In 2021, almost 8 000 persons were newly diagnosed with CRC in Belgium. In Flanders (58% of the Belgian population), the age-standardized incidence rates (world standard population) for CRC were 33.9 per 100,000 person-years for men and 24.2 per 100,000 person-years for women in 2021 (2).

Most CRC tumors slowly develop through multiple steps starting from precancerous lesions. Over time, both morphologic, histologic and also molecular modifications can accumulate, leading to invasive tumors. As for most cancer types, prognosis of CRC depends on the tumor stage at diagnosis, and can be drastically improved by early detection (3). This is clearly shown by the 5-year survival rates in Flanders, which are 96% for stage I and only 21% for stage IV in persons between 50 through 74 years old (4). Moreover, in high-income countries, incidence rates of CRC have been decreasing as a result of the implementation of screening programs. If these screening programs are effective and in place in all European countries, an additional 80 000 CRC deaths could be prevented yearly (5). It is clear that screening of CRC is an important tool to reduce incidence and mortality. In Flanders, CRC screening was introduced in October 2013 for all persons aged 56 through 74 years. The starting age was gradually lowered from 56 in 2013 to 50 in 2020. Currently, the program offers a biennial, free of charge fecal immunochemical test (FIT: FOB Gold, Sentinel Diagnostics, Milan, Italy) to individuals from 50 through 74 years old (6). More details about the program and the inclusion/exclusion criteria for screening have previously been described (7).

One of the important quality indicators to consider in the CRC screening program is the occurrence of FIT interval cancers (FIT-ICs). FIT-IC are defined as CRC diagnosed after a negative FIT, but before the next FIT invitation (24 months) (7, 8). On the other hand, screen-detected (SD) cancers are defined as a CRC diagnosed after a positive FIT, within 6 months after the first follow-up colonoscopy and before the next recommended FIT invitation (24 months) (7). Previous research by Tran et al. (7) about FIT-ICs already showed an overall FIT-IC proportion of 13%. Importantly, they also reported a significantly higher risk of having a FIT-IC versus an SDCRC for female gender, older age, right-sided and rectum location, high differentiation grade and stage IV compared to stage I participants in the in-study population, for which the latter showed the strongest association with the risk of having FIT-IC (OR= 7.15 [5.76 – 8.88]) When looking at stage IV alone, the FIT-IC proportion was 45% (7).

In addition to these findings, it has been described that relative 5-year survival rates for FIT-IC are drastically lower compared to SD-ICs (67% vs 94% respectively). The larger proportion of stage IV cancers in the FIT-IC group lead to this lower relative survival chances (9). Furthermore, FIT still has room for improvement in sensitivity, which is currently 79% (ranging from 70% – 86% in different studies) for stage IV (9). Specific and reliable markers for ICs can help here to increase the SD IV proportion.

Unfortunately, literature about clinicopathological and molecular features of FIT-IC cancers is rare, and large studies are still lacking. Therefore, we wanted to extend the previous study by Tran et al. (7), by exploring clinicopathological and molecular characteristics of FIT-IC versus FIT-SD CRC. Taking into account the results of Tran et al., suggesting that stage IV is the most interesting subgroup for further investigations, we expanded the existing data of all advanced stage (IV) patients with clinicopathological and molecular data. Since our stage IV patient group consists of n= 511 patients for which we manually extracted data, we chose to focus our study on this (most interesting) stage IV group. Our research objective was to model the associations between the risk of having an SD versus FIT-IC stage IV CRC and the identified (significantly) different characteristics, called variables hereafter.

## 2 Material and methods

### 2.1 Study population and study design

This retrospective population-based study is a follow-up study of Tran et al. (7). In the current study, all eligible individuals (between 53 through -74 years old) who participated in the Flemish CRC screening program between October 2013 (start of the program) and December 2018 (last year with all required data complete) and who were diagnosed with either a stage IV SD or FIT-IC CRC in the same period were selected. All cases were screened using another FIT (OC Sensor, Eiken, Japan). We adopt the same definitions as those used in the previous study for SD and FIT-IC CRC, where SD CRC was defined as “a CRC that was diagnosed within 6 months after the first follow-up colonoscopy for a positive FIT and before the next recommended FIT invitation (24 months)” (7). A FIT-IC CRC was defined as “a CRC diagnosed after a negative FIT and before the next recommended FIT invitation (24 months)”. Staging was performed using the applicable TNM edition at the time of diagnosis (TNM 7^th^ edition for incidence years 2013-2016, TNM 8^th^ edition starting from incidence year 2017). Combined TNM stage was a compilation of pathological (pTNM) and clinical (cTNM) stage. pTNM prevails over cTNM, except when cTNM stage is IV (10). All available clinical, clinicopathological and molecular data was extracted from the Belgian Cancer Registry (BCR).

### 2.2 Data sources and studied variables

Data from all patients (n=511) was obtained from several databases. First, the Flemish Centre for Cancer Detection provided data on participant’s screening history (FIT result and follow-up colonoscopy), which originates from linkage of data from the InterMutualistic Agency. Then, population-based data from the Belgian Cancer Registry (BCR) was subsequently used to link these data to personal information (gender, age), tumor characteristics (location, adenocarcinoma type…), clinicopathological and molecular features (lymphovascular invasion, genetic mutations…). All variables are described in **Table 1**. For patients with multiple lesions (<5% of the total population), the lesions were considered independent. Tumor location was classified as right sided colon (from cecum to hepatic flexure), transverse colon or left sided colon (from splenic flexure to the sigmoid). In our population, there were no patients with stage IV rectal tumors. Tumor types were split in adenocarcinomas (‘no specific type (NST)’, mucinous and signet ring cell carcinomas) and neuroendocrine tumors (NET). Tumor sizes were grouped per 2 cm. Tumor descriptions were summarized as bulky (including polypoid, bourgeois and stenosing tumors), ulcerative (comprising flat, ulcerating and infiltrating tumors) or both (combinations of the former). The presence or absence of another primary tumor was reported as such. In case a significant result was found, additional analysis for the location of the other primary tumor was performed. Lymphovascular invasion (LVI) was subdivided in being absent (none), only lymphatic invasion, only vascular invasion and both lymphatic/vascular invasion. Depth of Invasion (Di) was subdivided in T0-2, T3 and T4. Microsatellite instability (MSI) was categorized as stable, low or high. DNA mismatch repair genes were documented in view of the protein staining and were described as positive when a loss of staining was found.

**Table 1:** Study population characteristics.

Parameters	Outcome – split IC/SD		Total n
Outcome	**IC, n (%)**	**SD, n (%)**	
	215 (42.2%)	292 (57.6%)	507
Gender, female	112 (52.1%)	103 (35.3%)	507
Location			
Right	106 (51.2%)	66 (23.1%)	493
Left	85 (41.1%)	210 (73.4%)	
Transverse	16 (7.7%)	10 (3.5%)	
Age (years)			
Median (IQR)	66 (62-71)		507
50-54	0 (0.0%)	3 (1.0%)	
55-59	31 (14.4%)	43 (14.7%)	
60-64	45 (20.9%)	69 (23.6%)	
65-69	58 (27.0%)	82 (28.1%)	
70-74	60 (27.9%)	92 (31.5%)	
75-79	21 (9.8%)	3 (1.0%)	
Type of adenocarcinoma*			
NST	161 (75.6%)	247 (84.9%)	504
Mucinous	37 (17.4%)	33 (11.3%)	
Signet ring cell	15 (7.0%)	11 (3.8%)	
NET (presence)	12 (5.6%)	4 (1.4%)	505
Tumor size (cm)			
Median (IQR)	3.75 (2.35 – 5.75)		303
0 – 2.0	12 (12.5%)	33 (16.4%)	
2.1 – 4.0	51 (53.1%)	101 (50.2%)	
4.1 – 6.0	26 (27.1%)	50 (24.9%)	
6.1 – 8.0	5 (5.2%)	12 (6.0%)	
8.1 – 10.0	3 (3.1%)	3 (1.5%)	
10.1 – 12.0	3 (3.1%)	2 (1.0%)	
12.1 – 14.0	1 (1.0%)	0 (0.0%)	
16.1 – 18.0	1 (1.0%)	0 (0.0%)	
Tumour description			
Bulky	21 (22.1%)	23 (14.2%)	257
Ulcerative	61 (64.2%)	103 (63.6%)	
Both	13 (13.7%)	36 (22.2%)	
Lymph node metastasis (presence)	93 (77.5%)	151 (72.2%)	329
Lymphovascular invasion			
None	30 (25.6%)	85 (42.9%)	315
Only lymphatic invasion	10 (8.5%)	18 (9.1%)	
Only vascular invasion	15 (12.8%)	19 (9.6%)	
Both lymphatic and vascular invasion	62 (53.0%)	76 (38.4%)	
Depth of invasion			
T0-2	9 (7.8%)	23 (11.3%)	320
T3	55 (47.4%)	127 (62.3%)	
T4	52 (44.8%)	54 (26.5%)	
Perineurinal invasion (presence)	39 (39.8%)	62 (36.7%)	267
Extra tumoral deposits (presence)	31 (60.8%)	41 (55.4%)	125
MSI			
Stable	77 (95.1%)	101 (54.3%)	186
Low	2 (2.5%)	1 (0.9%)	
High	2 (2.5%)	3 (2.8%)	
MLH1 (positive)	105 (99.1%)	146 (97.3%)	259
PMS2 (positive)	104 (95.4%)	138 (97.2%)	251
MSH2 (positive)	106 (99.1%)	149 (100%)	256
MSH6 (positive)	106 (97.2%)	147 (100%)	256
MLH1 methylation (presence)	1 (100.0%)	1 (100.0%)	2
APC (positive)	11 (84.6%)	6 (85.7%)	20
KRAS (positive)	57 (47.1%)	89 (48.6%)	304
NRAS (positive)	8 (8.3%)	7 (5.2%)	230
HRAS (positive)	0 (0.0%)	0 (0.0%)	78
BRAF (positive)	16 (18.2%)	13 (13.1%)	187
PIK3CA (positive)	12 (27.9%)	4 (8.3%)	91
Other primary tumor (presence)	35 (16.8%)	35 (12.0%)	507

IC, Interval cancer; SD, screen-detected; IQR, inter quartile range; NST, no specific type; NET, neuro-endocrine tumor; MSI, microsatellite instability.

*Adenosquamous and medullary adenocarcinoma were left out because there were too few cases.

### 2.3 Statistical analyses

#### 2.3.1 Sample size and missing data

We included all 507 eligible stage IV CRCs diagnosed tumors in the Flemish CRC screening program between October 2013 and December 2018. For a few patients, there was no information about the tumor; these cases were removed. Data on gender, age and other primary tumors was complete (see **Table 1**). Data for location was complete for 97% and type of adenocarcinoma for 99% of all cases. **Table 1** gives an overview of the number of cases for which data on the different characteristics were available.

#### 2.3.2 Main analyses

All categorical variables were described as counts and percentages. Continuous variables were described with their median and interquartile ranges. To evaluate whether significant differences were present between SD or IC CRC per characteristic, we performed either a Welch’s t-test for continuous variables or a chi-square/fisher exact test for categorical variables. P-values below 0.05 (two-sided) were considered to be statistically significant. For variables consisting of multiple levels (e.g. type of adenocarcinoma), a Benjamini-Hochberg correction for multiple testing was implemented. P-values less than the adjusted threshold based on a 0.1 false discovery rate (FDR) were considered to be statistically significant. A higher FDR of 0.1 was used because of the exploratory character of the analyses. The Benjamini-Hochberg correction is demonstrated in **Supplementary Table 1**.

To identify variables associated with the odds of having a stage IV SD or FIT-IC CRC, logistic regression was performed. All variables that were significantly different in the exploratory analyses, were first tested in an univariate model. Crude odds ratios with 95% confidence intervals were reported. A likelihood ratio test was performed with null hypothesis that all categories carry the same odds to have a FIT-IC. Benjamini-Hochberg correction was applied to correct for multiple testing. P-values below the adjusted threshold based on a 0.05 false discovery rate (FDR) were considered to be statistically significant. In case the independent variable had more than two levels, *post hoc* analysis with Dunnett correction was performed.

The previous study by Tran et al. (7) found significant associations between age, gender, and location and the risk of having FIT-IC vs SD-CRC, therefore we included these variables as covariates in multivariable analyses in the current study. Multicollinearity between the covariates and other independent variables was checked and only reported if present. Adjusted odds ratios with 95% confidence intervals were reported. P-values below the adjusted threshold based on a 0.05 false discovery rate (FDR) were considered to be statistically significant. Benjamini-Hochberg correction is demonstrated in **Supplementary Table 2**.

### 2.4 Privacy, ethical approval and consent to participate

When participating in the Flemish CRC-SP, all participants filled out a written informed consent explaining that personal information can be used for scientific research and evaluation to improve the CRC screening program. In this study, data from the Flemish Centre for Cancer Detection and Belgian Cancer Registry was used, for which approval was given by the Belgian Privacy Commission [reference IVC/KSZG/19/236, number 13/091 (11)]. All data was pseudonymized. The study was conducted in accordance with the Declaration of Helsinki.

## 3 Results

### 3.1 Study population

A total of 507 stage IV CRCs were included, 215 (42.2%) of which were diagnosed with a FIT-IC and 292 (57.6%) with an SD CRC. Most of the FIT-ICs were diagnosed among females (52.1%) with right sided tumors (51.2%) at older age (70-74; 27.9%), as reported before (7). Overall, most of the tumors were adenocarcinomas with no specific type (NST, 81.0%). For 8 out of 27 variables, data was >90% complete. For 10 out of 27 variables, data was >50% complete. For 9 out of 27 variables, data was less than 50% complete. For 4 variables, data was less than 20% complete (see **Table 1**).

### 3.2 Exploratory analyses of clinicopathological and molecular features in SD vs FIT-IC CRC

**Table 2** gives an overview of all statistical analyses and the corresponding P-values. For gender, location, NET and presence of a *PIK3CA* mutation a significant difference in SD vs FIT-IC was found. For gender and location, this is in line with the previous study (7). NET and *PIK3CA* presence were more frequent for FIT-IC CRC. Adenocarcinoma type, depth of invasion and lymphovascular invasion also showed significant differences between SD and FIT-IC, and since these variables had multiple categories, further testing was performed. We found significant differences between T0-2 vs T4 and T3 vs T4 of SD vs FIT-IC CRC’s depth of invasion, where T4 was more frequent in FIT-ICs but T0-2 and T3 more frequent in SD CRC. Also, there was a significant difference found for (lympho)vascular invasion compared to no invasion for SD vs FIT-IC CRC. Here, lymphovascular invasion was more common in FIT-IC, while no invasion was more frequent in SD CRC. For type of adenocarcima, NST vs mucinous was significant for SD vs FIT-IC with mucinous adenocarcinoma being more frequent in FIT-ICs and adenocarcinomas of NST more frequent in SD CRC. Age, tumor size and MSH6 mutations only show a trend towards significance (p-value < 0.1).

**Table 2:** Exploratory analyses of SD vs FIT-IC colon cancers.

Variable	P-value	P-value *Post hoc* ^1^
Gender	**0.000219**	/
Age	0.0757	/
Location	**4.32E-12**	Right vs Left: **3.48 E-12** Left vs Transverse: **0.000572**
Adenoma type	**0.0291**	NST vs mucinous: **0.0355**
NET	**0.0152**	/
Tumor size	0.0790	/
Tumor description	0.106	/
LNM	0.359	/
Depth of invasion	**0.00352**	0-2 vs 4: **0.0367** T3 vs T4: **0.00142**
Perineurinal invasion	0.708	/
Lymphovascular invasion	**0.0159**	None vs only vascular: **0.0443** None vs both: **0.00192**
ETD	0.679	/
MSI	0.847	/
MLH1_meth	NA	/
MLH1	0.731	/
PMS2	0.494	/
MSH2	0.411	/
MSH6	0.0795	/
APC	1	/
KRAS	0.809	/
NRAS	0.435	/
HRAS	NA	/
BRAF	0.426	/
PIK3CA	**0.0270**	/
Another primary tumor	0.196	/

NST, no specific type; NET, neuro-endocrine tumor; LNM, lymph node metastasis; ETD, Extra Tumoral Deposits; MSI, microsatellite instability; NA, not applicable.

^1^Multiple testing correction (Benjamini-Hochberg) was used. Only significant p-values are reported in this table in bold. More details of the adjusted p-values can be found in **Supplementary Table 1**.

### 3.3 Clinicopathological and molecular features associated with the risk of having a FIT-IC vs SD CRC

#### 3.3.1 Univariable logistic regression

For each variable that was found to be significant in the exploratory analyses, the odds of having a FIT-IC vs SD CRC was modeled using a univariable logistic regression. **Table 3** gives an overview of all tested variables, with p-values and crude odds ratios. Within the univariate models, NET presence, lymphovascular invasion and *PIK3CA* mutation were significant. FIT-IC CRC have a 4-fold increased odds to be a NET tumor (OR= 4.26 [1.46 – 15.40]), a 2-fold increased odds to present with lymphovascular invasion (OR= 2.31 [1.36 – 3.98]) and a 4-fold increased odds to have a *PIK3CA* mutation compared to SDCRC (OR= 4.25 [1.34 – 16.40]). The mucinous subtype of adenocarcinoma and depth of invasion T4 gave a close to significant result compared to NST and T0-2 respectively (p < 0.1).

**Table 3:** Logistic regression for variables with univariate associations.

Characteristic	Category	N for IC	N for SD	Crude OR (95% CI)	P-value	N for aIC	N for aSD	aOR (95% CI)	P-value^1^
Type of adenocarcinoma	NST	161 (31.9%)	247 (49.0%)	ref	**0.0303**	155 (31.6%)	242 (49.4%)	ref	0.747
	Mucinous	37 (7.3%)	33 (6.5%)	1.72 [1.03 – 2.87]	0.0724	36 (7.3%)	32 (6.5%)	1.16 [0.66 – 2.04]	/
	Signet ring cell	15 (3.0%)	11 (2.2%)	2.09 [0.94 – 4.78]	0.1377	14 (2.9%)	11 (2.2%)	1.28 [0.53 – 3.15]	/
NET	Absence	202 (40.0%)	287 (56.8%)	ref	**0.00709**	194 (39.5%)	281 (57.2%)	ref	**0.00336**
	Presence	12 (2.4%)	4 (0.8%)	4.26 [1.46 – 15.40]	NA	12 (2.4%)	4 (0.8%)	5.29 [1.71 – 20.00]	NA
Depth of Invasion	T0-2	9 (2.8%)	23 (7.2%)	Ref	**0.00382**	7 (2.4%)	18 (6.3%)	Ref	0.137
	T3	55 (17.2%)	127 (39.7%)	1.10 [0.49 – 2.67]	0.939	51 (17.8%)	114 (39.7%)	1.00 [0.41 – 2.62]	/
	T4	52 (16.3%)	54 (16.9%)	2.46 [1.07 – 6.07]	0.0616	49 (17.1%)	48 (16.7%)	1.66 [0.63 – 4.56]	/
Lymphovascular invasion	No	30 (9.5%)	85 (27.0%)	Ref	**0.0144**	23 (9.4%)	66 (27.0%)	Ref	**0.0244**
	Only lymphatic	10 (3.2%)	18 (5.7%)	1.57 [0.64 – 3.74]	0.645	10 (4.1%)	15 (6.1%)	1.42 [0.54 – 3.55]	0.949
	Only vascular	15 (4.8%)	19 (6.0%)	2.23 [1.00 – 4.96]	0.127	7 (2.9%)	11 (4.5%)	1.49 [0.60 – 3.61]	0.696
	Both	62 (19.7%)	76 (24.1%)	2.31 [1.36 – 3.98]	**0.00623**	48 (19.7%)	64 (26.2%)	2.50 [1.41 – 4.52]	**0.0151**
PIK3CA	Absence	31 (34.1%)	44 (48.4%)	Ref	0.0130*	31 (34.1%)	44 (48.4%)	Ref	0.163
	Presence	12 (13.2%)	4 (4.4%)	4.25 [1.34 – 16.40]	NA	12 (13.2%)	4 (4.4%)	2.6 [0.68 – 11.20]	NA

Univariate models are displayed, as well as multivariate models corrected for at least age, gender and location.

^1^ P-values next to the reference level refer to a likelihood ratio test, with null hypothesis that all categories carry the same odds to have a FIT-IC. P-values in bold survive the multiple hypothesis correction (Benjamimi-Hochberg, **Supplementary Table 2**). In case the independent variable has more than two levels, the P-values next to the non-reference levels refer to the Dunnett-corrected P-values from the post hoc analysis, comparing the non-reference level to the reference.

NA, not applicable.

#### 3.3.2 Multivariable logistic regression

**Figure 1:**
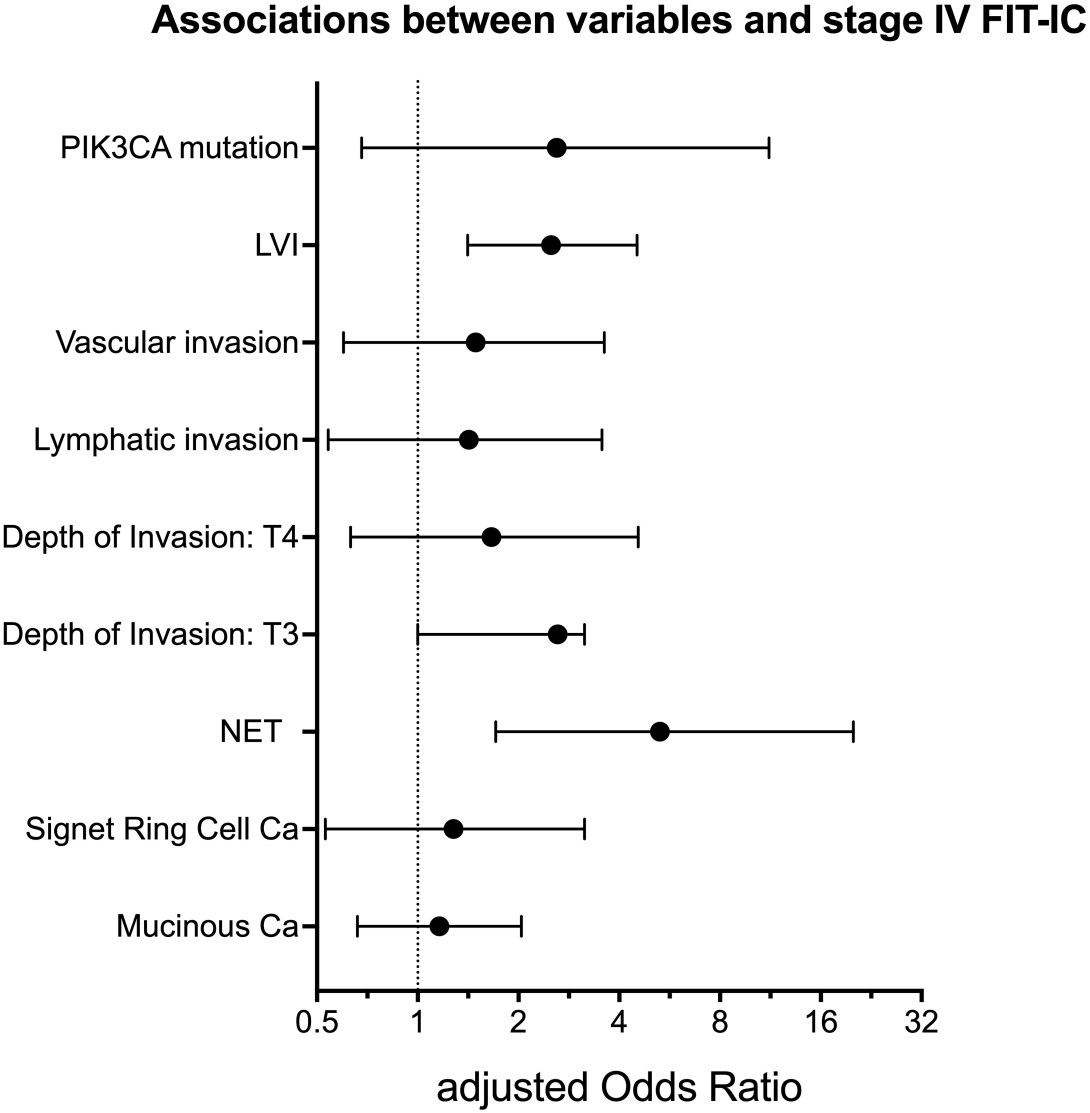
Adjusted Odds Ratio with 95% confidence intervals for variables associated with stage IV FIT-IC. LVI, lymphovascular invasion; NET, neuro-endocrine tumor; Ca, carcinoma. Figure created with Graphpad Prism v10.1.1.

Since the previous work showed associations between gender, age and location and the risk of having a FIT-IC vs SD CRC, we included these variables as covariates in the multiple logistic regression models (7). **Table 3** gives an overview of all tested variables, with p-values and adjusted odds ratios. **Figure 1** gives an overview of the adjusted odds ratios for the tested variables. In the multivariate models, only NET presence and lymphovascular invasion showed significant results. In the corrected models, FIT-IC CRC have 5 times higher odds to be a NET tumor (OR= 5.29 [1.71 – 20.00]) and 2.5 times higher odds to have lymphovascular invasion (OR= 2.50 [1.41 – 4.52]). All other variables completely lost significance when the models were corrected for age, gender and location.

## 4 Discussion

In this study, we identified differences between clinicopathological and molecular characteristics of SD and FIT-IC and assessed their associations to the risk of having a FIT-IC vs SD CRC.

In the exploratory analyses, we identified significant differences between SD versus FIT-IC for gender, presence of NET, depth of invasion (T3 vs T4), lymphovascular invasion (none vs both) and presence of a *PIK3CA* mutation. For ‘type of adenocarcinoma’, significant differences were found, but significance was lost when testing the different types separately, partly due to the Benjamini Hochsberg correction. Regression analyses of FIT-IC versus SD stage IV CRC revealed new interesting insights about the significant parameters and their association with higher risk of having a FIT-IC. All relevant factors are discussed below.

### 4.1 Previously found variables: gender, age and location

Consistent with the previous study by Tran et al. (7), there is a significant difference in prevalence of SD versus FIT-IC CRC between genders, with FIT-ICs more likely to occur in women. Reasons reported for this are a lower blood hemoglobin concentration, a longer colonic transit time and a higher proportion of right-sided tumors (7). It has been suggested that women are more likely to seek medical help when they experience symptoms, which may lead them to undergo diagnostic tests and, as a result, have their CRC diagnosed as FIT-IC (12, 13). Furthermore we replicated the very significant effect of location, especially right vs left. An in-depth discussion on these differences was given previously (7). In short, a longer transit time from the right side and higher proportion of flat tumors are thought to play a role herein. For these reasons, the current study included gender and location as covariates in the multiple regression analysis. Contrary to the previous study, we did not find a significant association between age nor age categories and the odds of having a FIT-IC versus a SD stage IV CRC. However, in this study we have only included stage IV SD and IC CRCs, which could explain these differences (7). Nevertheless, age was also taken into account as a covariate in later analyses (multiple logistic regression).

### 4.2 Tumor type

Within our study, we made the distinction between adenocarcinoma and NET colon tumors because of their different origin (epithelial vs neuroendocrine cells) and the rarity of NETs (14). Adenocarcinoma tumors were split by per histological type, as described by the WHO (15).

In literature, few studies have investigated adenocarcinoma type in SD vs FIT-ICs. Steel et al. (16) did report an association of aggressive histotypes (mucinous and signet ring cell adenocarcinoma) with higher risk of FIT-IC. However, in our exploratory analyses, we only found significant results for mucinous adenocarcinomas. Signet ring cell carcinoma lost its significance after Benjamini Hochsberg correction. Within the logistic regression analysis, mucinous adenocarcinoma was only found close to significant after correction for multiple testing. Importantly, the multiple logistic regression analysis revealed that, after adjusting the model for location, the type of adenocarcinoma was not significant anymore, indicating that location, especially right-sided tumor location, is an important variable that needs to be taken into account in the analysis of interval CRCs. It has been reported that mucinous adenocarcinomas occur in 10-20% of CRC patients, mostly women, in the proximal colon (right and transverse) (17). This is concordant with our population, where 13.6% cases were mucinous adenocarcinomas (both SD and FIT-IC), 57% of mucinous tumors being observed in female patients and 60% located in the right colon. Signet ring cell carcinoma was also reported to be more common in the proximal colon (18), which we also observed in our cohort (88.4% for both SD and FIT-IC). In all, our results show that tumor location is an important confounder, that needs to be accounted for in the analysis of the association between adenocarcinoma type and the odds of having a FIT-IC versus a SD CRC.

In both the exploratory and regression analyses, the presence of a NET tumor was found to be significantly different in FIT-IC vs SD CRC, with a 5.3 times higher odds of presence of NET in FIT-ICs. A few reasons could explain this observation. First, NET tumors are completely different compared to adenocarcinoma tumors in origin and clinical presentation (19). NETs tend to bleed less than adenocarcinomas (20), partly explaining the higher risk for FIT-IC, although 4 NETs were screen-detected in our cohort. However, we cannot be certain that for example, other lesions caused the bleeding and that the NET was accidently discovered during the colonoscopy This hypothesis was already described by others (21–23). However, if this had been the case, the number of SD NET would have been smaller and the number of interval NET would have been larger than the numbers shown in this study, leading to a larger OR which indicates a even stronger association between NET and the odds of being a FIT IC versus a SD CRC. In our study, we found 1.4% of all SD cancers to be NETs. In literature, there are very few research papers describing NETs found after colonoscopy. Most of those papers describe its prevalence, which is ranging from 0.018% in the English bowel screening program (21), to 0.16% in the Taiwanese program (24–27). However, these studies almost exclusively focus on rectal NETs, which we do not have in our cohort. Moreover, other studies take into account all stages. Therefore, it is difficult to compare our results to the existing literature. There is one study by Kim et al. that focuses on colon subepithelial tumors discovered by chance. Here, of the 105 detected tumors, 2 were NETs (1.9%), which is close to our rate of 1.4% (28).

Overall, it is important to keep in mind that colorectal NET tumors are difficult to find with FIT screening, and other options will need to be explored for NET detection in the colon.

### 4.3 Clinicopathological variables

Several clinicopathological variables were investigated in our study. They can be grouped by tumor-informed parameters (lymphovascular invasion (LVI), perineuronal invasion (PNI), depth of invasion (Di), lymph node metastasis (LNM) and extra tumoral deposits (ETD)) and tumor-agnostic parameters (microsatellite instability (MSI) and DNA mismatch repair (MMR) markers for MSI).

Regarding the tumor-informed parameters, only Di and LVI were found to be significant in our exploratory analysis. When further investigating these variables, we found associations between LVI and PNI, Di and LNM. Remarkably, Di was not associated with PNI. ETDs were not associated with LVI or Di. The associated variables were taken into account when performing the multiple regression analyses. In the final multivariable logistic regression analysis, only presence of LVI was found to be a significantly associated risk factor for FIT-IC vs SD, with almost 2 times higher odds of being a FIT IC versus a SD CRC. In literature, there is no information that describes the role of any of the clinicopathological variables in CRC FIT-ICs. The only relevant information is that LVI and PNI have been described as independent negative prognostic factors (i.e. poor outcome) in CRC development (29–31). There is one group that also describes LVI as a risk factor for developing metastasis in CRC (32), which underlines the importance of reporting LVI at diagnosis. Interestingly, presence of LVI was also described as a significantly factor in a study about SD vs IC in breast cancers, with LVI more present in IC (33). Together with our results, this shows an important role for LVI in several malignancies, but further research is needed to fully understand its role in (FIT-) ICs.

Regarding the tumor-agnostic clinicopathological features, we did not find any significant difference in our analyses. Only in the exploratory analysis, a positive MSH6 result was found to be close to significantly different in SD vs FIT-IC cancers (p= 0.08). In literature, there are only a few papers describing MSI in ICs, and the findings are contradictory. Agreeing with our findings, Soong et al. did not find any difference in MMR expression between IC and SD CRC (34). However, contrary to our research, their population was screened using colonoscopies and not FITs. The ICs were defined as “cancer detected in a diagnostic examination prior to the next recommended colonoscopy and at least 1 year after the last colonoscopy”. The gap between these screening tools might explain the differences found in the variables. A few other papers describe that, despite the insignificant results, MSI is observed to be more prevalent in ICs (35–37). We did not observe this (**Table 1**). Lastly, two research papers describe significant differences in MSI for SD vs IC, but they defined IC patients as “Subjects with 1 prior colonoscopy > 180 days before the diagnosis” and “individuals that had a complete colonoscopy performed within 5 years of the diagnosis of CRC” respectively, which is different from the definition in more recent literature (38, 39). Also, the sample size of the latter was small (n= 42) (39). There is clearly still no consensus about the role of MSI in (FIT-)ICs and despite the predictive role of MSI in CRC outcome (40), we cannot conclude if there is a role of MSI in the development of FIT-ICs.

### 4.4 Genetic alterations

The most commonly reported genes were investigated in our study. We found significant differences between SD and FIT-IC CRC for the presence of a *PIK3CA* mutation in the exploratory and close to significant results in the simple logistic regression analysis, with an OR of 4.25 in the latter.

When performing the multiple logistic regression analysis, the significance for *PIK3CA* was lost. In literature, there are no papers describing *PIK3CA* mutations in FIT-ICs. There are few papers describing *PIK3CA* mutations in a colonoscopy-screened population, where in concordance to our multivariate regression results, no significant differences were found between SD and IC CRC. However, sample sizes might have been too small in both our (for *PIK3CA*: n=92) and these other studies to detect less frequent genetic differences (34, 39, 41). Furthermore, several studies describe that *PIK3CA* mutations are more often present in right-sided colon tumors (42–45), which could explain why this variable lost significance after adjusting for tumor location. Nevertheless, the use of *PIK3CA* mutations as biomarkers remains a discussion point in literature. It has been described as a prognostic biomarker for aggressive tumor growth and increased risk of tumor recurrence (45). Furthermore, associations between FIT-IC and other genetic alterations in e.g. *KRAS* have been reported (45), although we did not find this in our analyses. All other genes that were studied in our cohort, did not show any significant difference between SD and FIT-IC CRCs. This is in line with what is described in literature (34, 39, 41, 46), although these studies do not involve FIT-screened patients but on colonoscopy-, flexible sigmoidoscopy- or gFOBT- screened patients.

### 4.5 Missing data, difficulties and limitations of this study

One of the difficulties of working with the pathology reports from different hospitals and different years (2013 to 2018) is the inconsistent reporting of different features. For some variables, such as tumor type, location, age and gender, the reports were almost complete (>97%), while for example the tumor-informed clinicopathological parameters were only complete for ~50% of all cases. This means different (sub)datasets had to be used for the analysis of each variable. In this view, we also performed the analyses with the subset of data where all variables were complete (total n= 247). Despite the loss of statistical significance in this analysis, similar trends were observed for NET, LVI and *PIK3CA*. We cannot distinguish if missing data is coming from inconsistent reporting or inconsistent testing. Although guidelines for testing e.g. MSI and molecular alterations do exist, implementation is not perfect. Moreover, there is no standardization of the pathology reports between Belgian hospitals. Lastly, additional testing, especially for molecular markers, is often reported in additional reports rather than added to the original report, where we extracted the data from. All these hurdles made it difficult to obtain 100% complete datasets in this study. However, missing is at random, therefore it is highly unlikely that this missingness would affect our conclusions.

For some parameters, e.g. *APC* and *MLH1* methylation, there was a lack of sufficient data for statistical analysis, as such the relevance of these alterations in FIT-IC could not be assessed. Overall, it was difficult to perform reliable analyses for molecular markers, mainly because of a small sample size for molecular alterations (only 18 – 60% data completeness). By the end of 2014, the European Society of Medical Oncology (ESMO) published evidence-based guidelines for molecular testing of specific genes of the *EGFR* pathway in metastatic CRC (47). This pathway contains important actionable targets for selection of first-line therapy (48, 49). Furthermore, *KRAS/NRAS* and *BRAF* are considered important predictive and prognostic biomarkers for treatment decisions in metastatic CRC (48). Our cohort data is partly coming from a period before this year, which can – to a certain extent – explain the lack of completeness in reporting of the mutations. However, this underlines the need to work towards more complete information. Up until today, there is still not enough evidence to use *PIK3CA* outside of clinical trials (49), which could explain why mutations in this gene are not frequently tested or reported. As a final remark on molecular alterations, the lack of significant findings in molecular alterations could also be because only stage IV patients were included. This lowered the number of cases and thus the chances to find significant associations with the study outcome, if there are any, for (less frequent) mutations.

### 4.6 Future perspectives

Despite the advantages of CRC screening there are still some important limitations in using FIT (50). For example, in Flanders, participation rates are suboptimal compared to levels recommended by the European commission (51), fluctuating between 48.0% and 52.5%, with a lower participation rate in the younger age groups (50-54 years). Around 1 in 5 non-responders have reported their reason for non-participation being either fear of a FIT false positive result or a dislike for the procedure of fecal testing (52). Therefore, other screening methods, including blood-based biomarkers, have gained more attention in recent years (53).

Very recently, the HUNT study of Brenne et al. showed that CRC can be detected up to 2 years prior to clinical diagnosis, based on methylated circulating tumor DNA (ctDNA) (54). This research suggests that patients could receive their diagnosis up to 2 years earlier than the clinical diagnosis if ctDNA analysis would be part of the CRC screening program. This also leads us to believe that FIT-IC proportions - particularly in stage IV - could potentially be lowered using ctDNA analysis. However, studies show that existing ctDNA-based tests, e.g. EpiProColon^®^ and Galleri^®^, are not cost-effective at their current cost ($192 and $950 respectively) and screening performance. Liquid biopsy testing for CRC could potentially become more cost-effective than FIT, but only if the cost is substantially lowered. Further clinical trials are needed to investigate also the uptake in large-scale population screening, as the current ctDNA tests are not yet suited for this purpose (45, 46).

In view of our results, liquid biopsy-based screening could for example indicate the presence of a NET tumor, based on NET-specific markers (55). Although rare, colorectal NETs are a tumor type that is often missed by the FIT and mostly detected by accident when performing a colonoscopy. In the future, larger analyses for molecular alterations could prove useful. Moreover, a trend towards epigenetic research could be followed by also investigating DNA methylation biomarkers for FIT-IC. In the future, more comprehensive reporting – albeit more consistent reporting or more consistent testing- should also be considered. Lastly, this research could be expanded towards all stages to find critical characteristics that could lead to diagnosis of earlier stage CRC.

## 5 Conclusion

In this study, we evaluated clinicopathological and molecular difference between SD vs FIT-IC stage IV CRC. Throughout all analyses, the presence of NET and lymphovascular invasion were newly identified as factors associated with higher odds of having a stage IV FIT-IC instead of a stage IV SD CRC. Further research will be needed to clarify how these insights might help in optimizing the Flemish CRC screening program. Besides these observations, we found that tumor location is a crucial covariate when analyzing clinicopathological and molecular factors in FIT-ICs. Therefore, tumor location should be taken into account (where applicable) in the analyses concerning FIT- ICs. Lastly, expanding the study to all stages and prospective validation of these and future results will be necessary before potentially implementing it into the program and as such, optimizing CRC screening.

## Data Availability

The data consists of sensitive information that must be kept in a strictly controlled environment of the Belgian Cancer Registry. All data was pseudonymized for this study. Requests to access these datasets should be directed toinfo@kankerregister.org.

## Supplementary material

Supplementary File 1 This file contains two supplementary tables, explaining the Benjamini-Hochberg *Post hoc* analysis (**Supplementary Table 1**) and Benjamini-Hochberg multiple testing correction for the logistic regression analyses (**Supplementary Table 2**).

## References

[1] Belgian Cancer Registry. Cijfers over kanker (2021). Available online at: https://kankerregister.org/Cijfers_over_kanker (Accessed on 19/01/2024).

[2] Belgian Cancer Registry. Cancer Fact Sheet Colorectal Cancer (2021). Available online at: https://kankerregister.org/media/docs/CancerFactSheets/2021/Cancer_Fact_Sheet_ColorectalCancer_2021.pdf (Accessed on 19/01/2024).

[3] Balchen V Simon K. Colorectal cancer development and advances in screening. Clin Interv Aging. (2016) 11:967–76. doi: 10.2147/CIA PMC4958365 27486317

[4] Centrum Voor Kankeropsporing SK. Het Bevolkingsonderzoek Dikke Darmkanker in Vlaanderen. CVKO, Belgium (2024).

[5] . Joint Statement Call for action on Colorectal Cancer Screening in the EU. Directorate-General for Health and Food Safety (2021).

[6] Hoeck S Pringels S Kellen E Van Herck K Martens P Van Limbergen E. First results of the Flemish colorectal cancer screening program: start-up- period late 2013. Acta Gastroenterol Belg. (2016) 79:421–8. 28209100

[7] Tran TN Peeters M Hoeck S Van Hal G Janssens S De Schutter H. Optimizing the colorectal cancer screening programme using faecal immunochemical test (FIT) in Flanders, Belgium from the “interval cancer” perspective. Br J Cancer. (2022) 126:1091–9. doi: 10.1038/s41416-021-01694-2 PMC8980044 35022524

[8] van de Veerdonk W Hoeck S Peeters M Van Hal G Francart J De Brabander I. Occurrence and characteristics of faecal immunochemical screen-detected cancers vs non–screen-detected cancers: Results from a Flemish colorectal cancer screening programme. United Eur Gastroenterol J. (2020) 8:185. doi: 10.1177/2050640619882157 PMC7079275 32213071

[9] Niedermaier T Balavarca Y Brenner H. Stage-specific sensitivity of fecal immunochemical tests for detecting colorectal cancer: systematic review and meta-analysis. Am J Gastroenterol Wolters Kluwer Health. (2020) p:56–69. doi: 10.14309/ajg.0000000000000465 PMC6946106 31850933

[10] Brierley JD Gospodarowicz MK Wittekind C. TNM Classification of Malignant Tumours, 8th Edition. Wiley-Blackwell (2016).

[11] Informatieveiligheidscomité - Kamer sociale zekerheid en gezondheid (2023). Available online at: https://www.ehealth.fgov.be/ehealthplatform/file/view/AWvhE2PqnF_Mkwg-mMCV?filename=13-091-n236-bevolkingsonderzoek%20dikkedarmkanker-gewijzigd%20op%202%20juli%202019.pdf (Accessed on 21/01/2024).

[12] Ballering AV Hartman TCO Verheij R Rosmalen JGM. Sex and gender differences in primary care help-seeking for common somatic symptoms: a longitudinal study. Scandinavian Journal of Primary Health Care (2024) 41(2):132–9. doi: 10.1080/02813432.2023.2191653 PMC10193899 36995265

[13] Evans REC Brotherstone H Miles A Wardle J. Gender differences in early detection of cancer(2024). Available online at: www.liebertpub.com (Accessed on 21/01/2024).

[14] Fleming M Ravula S Tatishchev SF Wang HL. Colorectal carcinoma: Pathologic aspects. J Gastrointest Oncol. (2012) 3:153–73. doi: 10.3978/j.issn.2078-6891.2012.030 PMC3418538 22943008

[15] WHO Classification of Tumours Editorial Board. Digestive System Tumours: WHO Classification of Tumours, 5th. (2019).

[16] Steel MJ Bukhari H Gentile L Telford J Schaeffer DF. Colorectal adenocarcinomas diagnosed following a negative faecal immunochemical test show high-risk pathological features in a colon screening programme. Histopathology. (2021) 78:710–6. doi: 10.1111/his.14278 33037645

[17] Luo C Cen S Ding G Wu W. Mucinous colorectal adenocarcinoma: Clinical pathology and treatment options. Cancer Commun BioMed Cent Ltd. (2019) 39(1):13. doi: 10.1186/s40880-019-0361-0 PMC6440160 30922401

[18] An Y Zhou J Lin G Wu H Cong L Li Y. Clinicopathological and molecular characteristics of colorectal signet ring cell carcinoma: A review. Pathol Oncol Res. (2021) 27:1609859. doi: 10.3389/pore.2021.1609859 34381313 PMC8351516

[19] Osagiede O Habermann E Day C Gabriel E Merchea A Lemini R. Factors associated with worse outcomes for colorectal neuroendocrine tumors in radical versus local resections. J Gastrointest Oncol. (2020) 11:836–46. doi: 10.21037/jgo PMC7657829 33209480

[20] Cancer center. colorectal cancer types (2022). Available online at: https://www.cancercenter.com/cancer-types/colorectal-cancer/types (Accessed on 23/01/2024).

[21] Basuroy R O’Donnell CM Srirajaskanthan R Ramage JK. Ileocolonic neuroendocrine tumours identified in the English bowel cancer screening programme. Colorectal Dis. (2018) 20:O85–91. doi: 10.1111/codi.14033 29368418

[22] Kooyker AI Verbeek WHM van den Berg JG Tesselaar MET van Leerdam ME. Change in incidence, characteristics and management of colorectal neuroendocrine tumours in the Netherlands in the last decade. United Eur Gastroenterol J. (2020) 8:59–67. doi: 10.1177/2050640619865113 PMC7006007 32213058

[23] Vootla V Ahmed R Niazi M Balar B Nayudu S. Synchronous adenocarcinoma of the colon and rectal carcinoid. Case Rep Gastroenterol. (2016) 10:600–4. doi: 10.1159/000450677 PMC5121549 27920648

[24] Lin C Chung C Chiang T Tu C Liang C. Detection of rectal neuroendocrine tumor during screening colonoscopy and its difference from colonic adenocarcinoma. Adv Digestive Med. (2017) 4:99–104. doi: 10.1002/aid2.12099

[25] Kooyker AI Verbeek WH van den Berg JG Tesselaar ME van Leerdam ME. Change in incidence, characteristics and management of colorectal neuroendocrine tumours in the Netherlands in the last decade. United Eur Gastroenterol J. (2020) 8:59–67. doi: 10.1177/2050640619865113 PMC7006007 32213058

[26] Nagtegaal ID Vink-Börger E Kuijpers CCHJ Dekker E Shepherd NA. Incidental findings in the bowel cancer population screening program: other polyps and Malignancies – A nationwide study. Histopathology. (2023) 82:254–63. doi: 10.1111/his.14805 PMC10092619 36156277

[27] Gallo C Rossi RE Cavalcoli F Barbaro F Boškoski I Invernizzi P. Rectal neuroendocrine tumors: Current advances in management, treatment, and surveillance. World J Gastroenterol. (2022) 28:1123–38. doi: 10.3748/wjg.v28.i11.1123 PMC8985485 35431507

[28] Kim A Hong SN Chang DK Kim Y-H Kim JE Kim ER. Clinicopathologic and endosonographic characteristics of colon subepithelial tumors discovered incidentally. Diagnostics. (2024) 14:551. doi: 10.3390/diagnostics14050551 38473024 PMC10930823

[29] Bianchi G Annicchiarico A Morini A Pagliai L Crafa P Leonardi F. Three distinct outcomes in patients with colorectal adenocarcinoma and lymphovascular invasion: the good, the bad, and the ugly. Int J Colorectal Dis. (2021) 36:2671–81. doi: 10.1007/s00384-021-04004-7 PMC8589793 34417853

[30] Al-Sukhni E Attwood K Gabriel EM LeVea CM Kanehira K Nurkin SJ. Lymphovascular and perineural invasion are associated with poor prognostic features and outcomes in colorectal cancer: A retrospective cohort study. Int J Surgery. (2017) 37:42–9. doi: 10.1016/j.ijsu.2016.08.528 27600906

[31] Yahyazadeh H Mafi AR Khatooni E Beheshti M Abdollahinejad A. Lymphovascular and perineural invasions are independently associated with advanced colorectal carcinoma. Int J Cancer Manage. (2019) 12. doi: 10.5812/ijcm

[32] Rönnow C-F Arthursson V Toth E Krarup P-M Syk I Thorlacius H. Lymphovascular infiltration, not depth of invasion, is the critical risk factor of metastases in early colorectal cancer. Ann Surg. (2022) 275:e148–54. doi: 10.1097/SLA.0000000000003854 32187031

[33] Pálka I Kelemen G Ormándi K Lázár G Nyári T Thurzó L. Tumor characteristics in screen-detected and symptomatic breast cancers. Pathol Oncol Res. (2008) 14:161–7. doi: 10.1007/s12253-008-9010-7 18347932

[34] Soong TR Nayor J Stachler MD Perencevich M Jajoo K Saltzman JR. Clinicopathologic and genetic characteristics of interval colorectal carcinomas favor origin from missed or incompletely excised precursors. Modern Pathol. (2019) 32:666–74. doi: 10.1038/s41379-018-0176-6 30455417

[35] van der Vlugt M Carvalho B Fliers J Montazeri N Rausch C Grobbee EJ. Missed colorectal cancers in a fecal immunochemical test-based screening program: Molecular profiling of interval carcinomas. World J Gastrointest Oncol. (2022) 14:2195–207. doi: 10.4251/wjgo.v14.i11.2195 PMC9694267 36438700

[36] Lee YM Huh KC. Clinical and biological features of interval colorectal cancer. Clin Endosc Korean Soc Gastrointestinal Endoscopy. (2017) p:254–60. doi: 10.5946/ce.2016.115 PMC5475521 28320200

[37] Dong SH Huang JQ Chen JS. Interval colorectal cancer: A challenging field in colorectal cancer. Future Oncol Future Med Ltd. (2018) p:1307–16. doi: 10.2217/fon-2017-0439 29741114

[38] Stoffel EM Erichsen R Frøslev T Pedersen L Vyberg M Koeppe E. Clinical and molecular characteristics of post-colonoscopy colorectal cancer: A population-based study. Gastroenterology. (2016) 151:870–878.e3. doi: 10.1053/j.gastro.2016.07.010 27443823 PMC5159224

[39] Richter JM Pino MS Austin TR Campbell E Szymonifka J Russo AL. Genetic mechanisms in interval colon cancers. Dig Dis Sci. (2014) 59:2255–63. doi: 10.1007/s10620-014-3134-2 24705641

[40] Gryfe R Kim H Hsieh ETK Aronson MD Holowaty EJ Bull SB. Tumor microsatellite instability and clinical outcome in young patients with colorectal cancer. New Engl J Med. (2000) 342:69–77. doi: 10.1056/NEJM200001133420201 10631274

[41] Yang K Cao Y Gurjao C Liu Y Guo CG Lo CH. Clinical and genomic characterization of interval colorectal cancer in 3 prospective cohorts. Gastroenterology. (2022) 163:1522–1530.e5. doi: 10.1053/j.gastro.2022.08.020 35970241 PMC9691567

[42] Ahn AR Kim KM Jang KY Moon WS Ha GW Lee MR. Correlation of PIK3CA mutation with programmed death ligand-1 (PD-L1) expression and their clinicopathological significance in colorectal cancer. Ann Transl Med. (2021) 9:1406–6. doi: 10.21037/atm PMC8506770 34733958

[43] Yamauchi M Morikawa T Kuchiba A Imamura Y Qian ZR Nishihara R. Assessment of colorectal cancer molecular features along bowel subsites challenges the conception of distinct dichotomy of proximal versus distal colorectum. Gut. (2012) 61:847–54. doi: 10.1136/gutjnl-2011-300865 PMC3345105 22427238

[44] Rosty C Young JP Walsh MD Clendenning M Sanderson K Walters RJ. PIK3CA activating mutation in colorectal carcinoma: associations with molecular features and survival. PloS One. (2013) 8. doi: 10.1371/journal.pone.0065479 PMC3681782 23785428

[45] Vacante M Borzì AM Basile F Biondi A. Biomarkers in colorectal cancer: Current clinical utility and future perspectives. World J Clin Cases Baishideng Publishing Group Co. (2018) p:869–81. doi: 10.12998/wjcc.v6.i15.869 PMC6288499 30568941

[46] Ibáñez-Sanz G Sanz-Pamplona R Garcia M Beaulieu J-F Binefa G. Future prospects of colorectal cancer screening: characterizing interval cancers. Cancers (Basel). (2021) 13:1328. doi: 10.3390/cancers13061328 33809520 PMC8001713

[47] Van Cutsem E Cervantes A Nordlinger B Arnold D The ESMO Guidelines Working Group. Metastatic colorectal cancer: ESMO clinical practice guidelines for diagnosis, treatment and follow-up. Ann Oncol. (2014) 25:iii1–9. doi: 10.1093/annonc/mdu260 25190710

[48] Sepulveda AR Hamilton SR Allegra CJ Grody W Cushman-Vokoun AM Funkhouser WK. Molecular biomarkers for the evaluation of colorectal cancer. J Mol Diagnostics. (2017) 19:187–225. doi: 10.1016/j.jmoldx.2016.11.001 PMC5971222 28185757

[49] Cervantes A Adam R Roselló S Arnold D Normanno N Taïeb J. Metastatic colorectal cancer: ESMO Clinical Practice Guideline for diagnosis, treatment and follow-up ☆. Ann Oncol. (2023) 34:10–32. doi: 10.1016/j.annonc.2022.10.003 36307056

[50] Niederreiter M Niederreiter L Schmiderer A Tilg H Djanani A. Colorectal cancer screening and prevention—pros and cons. Memo Magazine Eur Med Oncol Springer-Verlag Wien. (2019) p:239–43. doi: 10.1007/s12254-019-00520-z

[51] Segnan N Patnick J Karsa Lv European Commission. Directorate General for Health & Consumers., International Agency for Research on Cancer. European guidelines for quality assurance in colorectal cancer screening and diagnosis. Publications Office of the European Union (2010). doi: 10.2772/1458

[52] Hoeck S Tran TN. Self-reported reasons for inconsistent participation in colorectal cancer screening using FIT in Flanders, Belgium. Gastrointestinal Disord. (2023) 5:1–14. doi: 10.3390/gidisord5010001

[53] Ferrari A Neefs I Hoeck S Peeters M Van Hal G. Towards novel non-invasive colorectal cancer screening methods: A comprehensive review. Cancers (Basel). (2021) 13:1820. doi: 10.3390/cancers13081820 33920293 PMC8070308

[54] Brenne SS Madsen PH Pedersen IS Hveem K Skorpen F Krarup HB. Colorectal cancer detected by liquid biopsy 2 years prior to clinical diagnosis in the HUNT study. Br J Cancer. (2023) 129:861–8. doi: 10.1038/s41416-023-02337-4 PMC10449868 37438612

[55] Mariën L Islam O Chhajlani S Lybaert W Peeters M Van Camp G. The quest for circulating biomarkers in neuroendocrine neoplasms: a clinical perspective. Curr Treat Options Oncol. (2023) 24(12):1833–51. https://link.springer.com/10.1007/s11864-023-01147-3. 10.1007/s11864-023-01147-3 37989978

