# Supplementary material for: Clinicopathological and molecular differences between stage IV screen-detected and interval colorectal cancers in the Flemish screening program
Source: Front Oncol. 2024 Sep 2;14:1409196. doi: 10.3389/fonc.2024.1409196 (PMC11402608; doi:10.3389/fonc.2024.1409196)
Supplement: Supplementary File 1 — This file contains two supplementary tables, explaining the Benjamini-Hochberg Post hoc analysis ( Supplementary Table 1 ) and Benjamini-Hochberg multiple testing correction for the logistic regression analyses ( Supplementary Table 2 ). [file Table1.docx]

**Supplementary Table 1: Adjusted P-values from the Benjamini – Hochberg Post-hoc analysis of the exploratory analysis**

| **Characteristic** | **P value** | **Rank (i)** | **Tests performed (m)** | **FDR (Q)** | **(i/m)*Q** |
| --- | --- | --- | --- | --- | --- |
| Right vs Left | 3.47E-12 | 1 | 15 | 0.1 | 0.0067 |
| Left vs Transverse | 0.000572 | 2 | 15 | 0.1 | 0.0133 |
| T3 vs 4T | 0.00142 | 3 | 15 | 0.1 | 0.0200 |
| No LVI vs LVI | 0.00192 | 4 | 15 | 0.1 | 0.0267 |
| NST vs Mucinous Ca | 0.0355 | 5 | 15 | 0.1 | 0.0333 |
| T0-2 vs T4 | 0.0366 | 6 | 15 | 0.1 | 0.0400 |
| No LVI vs only vascular | 0.0443 | 7 | 15 | 0.1 | 0.0467 |
| NST Ca vs signet ring cell Ca | 0.0664 | 8 | 15 | 0.1 | 0.0533 |
| No LVI vs only lymphatic | 0.309 | 9 | 15 | 0.1 | 0.0600 |
| Only lymphatic vs LVI | 0.370 | 10 | 15 | 0.1 | 0.0667 |
| Only llymphatic vs only vascular invasion | 0.502 | 11 | 15 | 0.1 | 0.0733 |
| Mucinous Ca vs signet ring cell Ca | 0.677 | 12 | 15 | 0.1 | 0.0800 |
| T0-2 vs T3 | 0.811 | 13 | 15 | 0.1 | 0.0867 |
| Vascular vs LVI | 0.932 | 14 | 15 | 0.1 | 0.0933 |
| Right vs Transverse | 0.993 | 15 | 15 | 0.1 | 0.1000 |

M= number of comparisons performed (15). Q= False Discovery Rate (0.1). LVI= lymphovascular invasion. NST= no special type. Ca= Carcinoma

**Supplementary Table 2: Adjusted P-values from the Benjamini – Hochberg correction of the logistic regression analyses**

| **Analysis** | **Characteristic** | **P value** | **tests performed (m)** | **Rank (i)** | **FDR (Q)** | **(i/m)*Q** |
| --- | --- | --- | --- | --- | --- | --- |
| Multivariate | NET | 0.00336 | 10 | 1 | 0.05 | 0.0050 |
| Univariate | Depth of Invasion | 0.00382 | 10 | 2 | 0.05 | 0.01000 |
| Univariate | NET | 0.00709 | 10 | 3 | 0.05 | 0.01500 |
| Univariate | PIK3CA mutation | 0.0130 | 10 | 4 | 0.05 | 0.0200 |
| Univariate | Lymphovascular invasion | 0.0144 | 10 | 5 | 0.05 | 0.0250 |
| Multivariate | Lymphovascular invasion | 0.0244 | 10 | 6 | 0.05 | 0.0300 |
| Univariate | Type | 0.0303 | 10 | 7 | 0.05 | 0.0350 |
| Multivariate | Depth of Invasion | 0.137 | 10 | 8 | 0.05 | 0.0400 |
| Multivariate | PIK3CA mutation | 0.163 | 10 | 9 | 0.05 | 0.0450 |
| Multivariate | Type | 0.747 | 10 | 10 | 0.05 | 0.0500 |

M= number of comparisons performed (30). Q= False Discovery Rate (0.05). LVI= lymphovascular invasion. NST= no special type. Ca= Carcinoma
